# Percutaneous solutions for biliary stent dysfunction: pictorial essay

**DOI:** 10.1590/0100-3984.2019.0141

**Published:** 2021

**Authors:** Thiago Franchi Nunes, Tiago Kojun Tibana, Gustavo Henrique Vieira de Andrade, Raphael Braz Levigard, Felipe Diniz Nogueira, Denis Szejnfeld

**Affiliations:** 1 Hospital Universitário Maria Aparecida Pedrossian da Universidade Federal de Mato Grosso do Sul (HUMAP-UFMS), Campo Grande, MS, Brazil.; 2 Hospital da Restauração, Recife, PE, Brazil.; 3 Radiologia Intervencionista Vascular e Oncológica (RIVOA), Rio de Janeiro, RJ, Brazil.; 4 Escola Paulista de Medicina da Universidade Federal de São Paulo (EPM-Unifesp), São Paulo, SP, Brazil.

**Keywords:** Radiology, interventional, Stents, Cholestasis/therapy, Radiologia intervencionista, Stents, Colestase/terapia

## Abstract

Plastic and metal biliary stents can fail to function properly, such failure being due to a positioning error or to the migration, occlusion, or fracture of the stent. An obstructed biliary stent can act as a nidus, causing complications such as recurrent persistent cholangitis. It can also cause vascular complications (such as bleeding and the formation of pseudoaneurysms), perforate the liver capsule (causing biloma or abscess), or, in rare cases, cause intestinal obstruction or perforation. In this pictorial essay, we demonstrate various interventional radiology techniques for the treatment of biliary stent dysfunction in patients with obstructive biliary disease.

## Abstract (pt)

Disfunção das próteses biliares plásticas ou metálicas pode ser causada por migração, oclusão, mau posicionamento ou fratura. Uma prótese disfuncional na via biliar pode atuar como *nidus* causando complicações como colangite recorrente e persistente. Pode ainda causar complicações vasculares como formação de pseudoaneurismas ou sangramento, e além disso, perfurar a cápsula hepática causando biloma ou abscesso, ou raramente, causar obstrução intestinal e/ou perfuração. Demonstramos diferentes técnicas da radiologia intervencionista no tratamento de endopróteses biliares plásticas e metálicas disfuncionais, em pacientes com doença biliar obstrutiva.

## INTRODUCTION

A series of recently published studies conducted in Brazil have highlighted the importance of interventional radiology in the diagnosis and treatment of various diseases^(1-5)^. Devices such as percutaneous transhepatic biliary drains, plastic biliary stents, and metal biliary stents are widely used to alleviate biliary obstruction in patients with inoperable tumors and in those with benign, postinflammatory, or iatrogenic stenosis. Such devices are inserted in the location of the stenosis with minimally invasive procedures either via endoscopic retrograde cholangiopancreatography (ERCP) or via ultrasound- or fluoroscopy-guided percutaneous transhepatic approaches^(6)^.

Biliary drains and stents occasionally malfunction because of occlusion, migration, or improper positioning, and various complications can occur if the failing stent or drain is not removed^(6,7)^. Complications related to drainage include occlusion, migration, improper positioning and, on rare occasions, catheter failure^(8)^.

Intervention with minimally invasive ERCP or a percutaneous approach are the initial options, open surgery being performed only when those procedures fail. In extremely rare cases when rigid catheters used for percutaneous transhepatic biliary drainage are fractured, the percutaneous method can be the ideal solution to recover the fragment and prevent further complications^(6)^.

The percutaneous transhepatic approach can be an alternative when the endoscopic intervention or reintervention fails. The first step is to perform percutaneous biliary drainage, after which the steps required in order to dislodge or remove the defective stent should be evaluated before a metal biliary stent is inserted. The percutaneous technique can significantly reduce the morbidity and mortality in the management of failing endoscopic biliary stents. Although various techniques for the management of failing stents have been described in the literature^(6-10)^, there is no clear evidence that any one technique is superior to any other. Our study demonstrates different techniques in the approach to biliary stent dysfunction. Although many complications have been described in the literature^(6-8)^, we have not observed any major complications associated with the techniques evaluated.

## PERCUTANEOUS SOLUTIONS

### Failing plastic biliary stents

**Figure 1:**
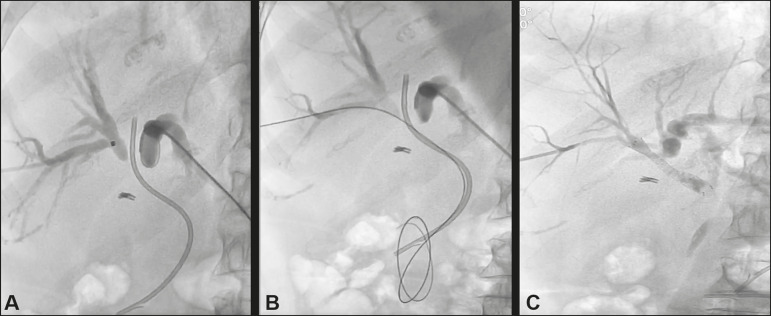
A 60-year-old female patient with Bismuth type IV cholangiocarcinoma underwent an endoscopic procedure for the implant of a 7 Fr plastic biliary stent. At 10 weeks after the procedure, her jaundice worsened. Computed tomography of the abdomen showed marked dilatation of the intrahepatic biliary tract due to improper positioning of the biliary stent. Percutaneous drainage and bilateral cholangiography (**A**) showed the failing plastic biliary stent. The stent was dislodged with a diagnostic catheter (**B**) and a new 6 × 60 mm biliary stent was placed in the right pre-papillary biliary tract (**C**). It was not possible to resolve the stenosis in the left biliary tract, and unilateral drainage was therefore performed. Her bilirubin levels normalized by day 30.

The percutaneous approach, via an established transhepatic route, is a safe option that also spares patients the discomfort of endoscopic procedures^(6)^. Studies of the use of percutaneous techniques have focused mainly on plastic biliary stents, describing various techniques to dislodge and push the fragment into the intestinal loops^(6-10)^, as depicted in Figure 1. During the percutaneous procedure, it is recommended to use a sheath to protect the hepatic parenchyma against the trauma of the sharp edges of the fractured catheter. In addition, an unobstructed pathway is necessary to reestablish the biliary drainage after removal of the fragment^(6,7)^.

**Figure 2:**
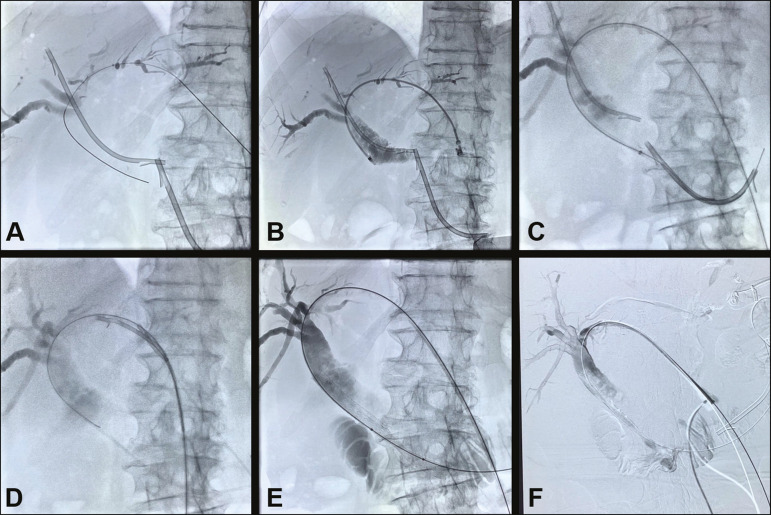
Patient with pancreatic neoplasia in whom a plastic biliary stent was placed via ERCP and developed an occlusion. A new attempt was made by first moving the old stent upward and then inserting a new one adjacently (**A**), although it did not result in an improvement in the jaundice or cholangitis. A percutaneous approach was used in order to position the new (distal) plastic biliary stent within the duodenum (**B,C**) and the old (proximal) stent was removed with a snare (**D**). A metal biliary stent was then put in place (E,F), resulting in improvement of the clinical and biochemical parameters.

When using a snare (Figure 2), the free peripheral end of the fragment of the fractured catheter is captured with the loop and firmly gripped, the fragment then being removed by using continuous traction^(1,2)^. When a balloon catheter is used, a guidewire is introduced into the percutaneous transhepatic tract and positioned within the fragment. The balloon can then be partially or completely inflated and pulled through the tract. However, this technique is highly operator dependent and takes more time, thus increasing the level of radiation to which the patient and the operator are exposed^(6)^.

### Relocation to the duodenal lumen

Various techniques have been described for moving a failing plastic biliary stent into the intestine (Figure 2). One well-known technique is repositioning through the use of a balloon catheter^(7)^. Diagnostic catheters or biliary drainage catheters can also be used^(6,11)^. The procedure can also be safely performed using a rigid wire, and the stent will typically pass through the intestine with no repercussions, although a few complications have been reported^(12)^. In a sample of patients with malignant obstructive jaundice, Gümüs^(7)^ observed no complications due to the procedure. Diagnostic angiographic catheters and biliary drainage catheters can be used in some cases, with no need to inflate a balloon, which is also an effective, economical repositioning method, or an additional catheter balloon can be used. 

### Removal through percutaneous transhepatic access

Percutaneous transhepatic removal of a plastic biliary stent can be performed by using a snare^(11)^ or by the modified technique described by Gümüs^(7)^, as illustrated in Figure 2. The Gümüs technique can be performed within the biliary tree or in the duodenum after the repositioning of the stent through the use of a long introducer sheath or a guide catheter^(7)^. Some authors have concluded that it is safer to perform these maneuvers in the duodenum (after the repositioning) than in the biliary tree^(7,13)^.

### Failing metal biliary stents

When metal biliary stents occlude, it is usually because of a sludge deposit or the growth of intraluminal tumor tissue. The sludge can be removed with a Fogarty balloon, whereas the internal tumor growth can be resolved with the insertion of a second biliary stent, either metal or plastic, depending on the life expectancy of the patient.

Breakage or fracture of a metal biliary stent often results in the intrahepatic retention of the catheter fragments and requires immediate action. In such cases, the ERCP techniques commonly used in the management of plastic biliary stents are typically not applicable, because the drainage catheter fragments are rigid and large, with sharp edges, which complicates their passage through the intestinal tract. If such a fragment were pushed into the intestinal loops, as would occur with a plastic biliary stent, the rate of complications, such as intestinal obstruction, perforation, and fistula formation^(6)^, would be higher.

In periampullary biliary obstructions, percutaneous drainage via ERCP is the first-line option for implanting stents for the clearance of biliary obstruction^(14)^. However, it is essential to pay attention to the technique used, to the arsenal of materials, and to the appropriate equipment at the time of the procedure. The success of the examination can hinge on a good preprocedure evaluation of the imaging examinations, identifying the extent of the lesion, determining whether the lesion involves any vascular structures, and assessing the degree of tumor necrosis. It is noteworthy that in the cholangiography of the obstructed biliary tract there is contrast stasis, which keeps the tract opaque for some time during fluoroscopy. However, in arteriography and venography, because of the high blood flow, there is no contrast stasis and the contrast medium delineating the blood vessels disappears very quickly under radioscopy. Locally extensive periampullary tumors typically also involve the portal vein. In such cases, ERCP catheterization can create access to the portal vein rather than the intrahepatic biliary tract, creating a portal vein fistula to the duodenum, which leads to a severe profile of hemorrhagic shock and cholangitis.

### Parallel placement

**Figure 3:**
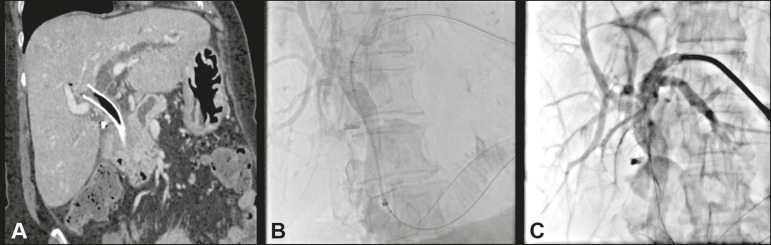
An 80-year-old female patient, with locally advanced adenocarcinoma of the pancreatic head, who presented with cholangitis. She underwent an endoscopic procedure to implant a metal stent (WallFlex; Boston Scientific, Marlborough, MA, USA). At two weeks after the procedure, her jaundice worsened. Computed tomography of the abdomen revealed marked dilatation of the left intrahepatic biliary tract-the consequence of an improperly positioned biliary stent, selective in the right hepatic duct by upward migration, occluding the confluence with the left hepatic duct (**A**). Another endoscopic procedure was performed to remove or reposition the stent. After its distal end had been captured, it was possible to crack the ring and the coating of the stent without moving it. Cholangiography showed peri-stent contrast retention and positioning of a different metal stent (Viabil; W.L. Gore, Flagstaff, AZ, USA) parallel to the WallFlex stent (**B**). Postprocedure cholangiography showed satisfactory biliary drainage into the duodenum (**C**).

Parallel metal biliary stent placement (Figure 3) in cases of previous occlusion can be performed with “side-by-side” or “stent-in-stent” insertion; the latter configuration is complex, the placement of more than two stents is rarely reported, and retreatment is challenging or impossible^(15)^. “Side-by-side” stent placement is the preferred configuration, because it allows reocclusion to be corrected more easily, especially in cases of transpapillary stent placement^(16)^.

### Perforating a covered stent

**Figure 4:**
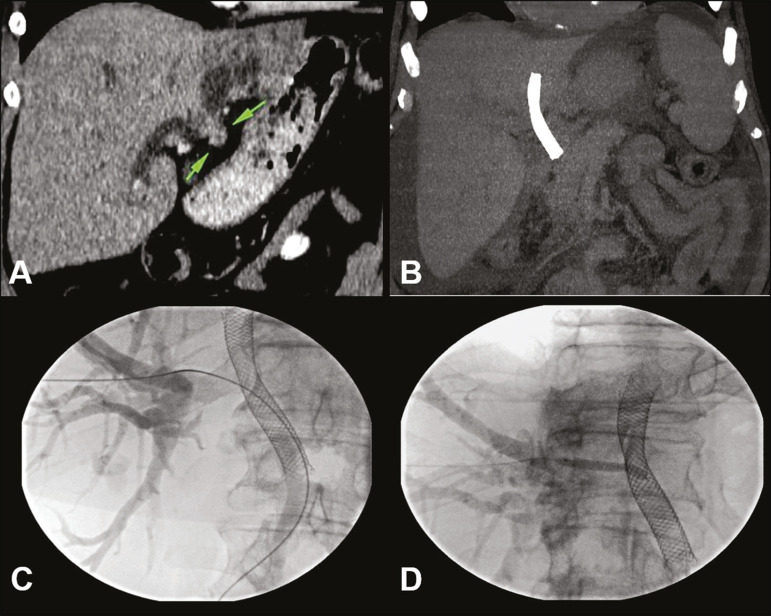
A 71-year-old male patient presenting with obstructive jaundice due to neoplasia (**A**). A coated, covered metal biliary stent was placed via ERCP. However, the stent placement did not result in an improvement in the obstruction. Subsequently, ERCP was again performed in order to place another (fenestrated) stent within the previously positioned stent. Computed tomography showed the original stent positioned in the left biliary tract (atrophied hepatic segment) and increased biliary dilatation in the right hepatic lobe (**B**). Because of the cholangitis, external percutaneous drainage of the right biliary tract was performed as an emergency procedure. After 60 days, the patient underwent the removal of the external drain and the RUPS-100 kit was used in order to introduce a hydrophilic wire into the original stent (**C**). Direct percutaneous puncture of the coated biliary stent was performed with the RUPS-100 cutting needle, after which a 5 × 40 mm expanding coronary balloon stent was positioned (**D**). The procedure resulting in technical success with no complications.

The placement of metal biliary stents in cases of previous occlusion of a totally covered stent seems quite challenging, because of the technical difficulty in passing a parallel second stent “side-by-side”, and especially a “stent-in-stent” insertion, because of the difficulty in passing the guidewire through the covering mesh. In these cases, an option would be to perform a “stent-in-stent” insertion with a transjugular liver access kit (RUPS-100; Cook Medical Inc., Bloomington, IN, USA) to perforate the covered biliary stent mesh (Figure 4).

### Removal of a stent and insertion of a new stent with a combined technique

The removal of a metal biliary stent inserted inadvertently between the duodenum and the portal vein is challenging and requires multidisciplinary collaboration, with access through the use of a combined technique such as the rendezvous technique. Technological advances-especially technical advances in interventional radiology-have favored the success of the procedure to remove porto-duodenal stents and insert new stents: one in the portal vein for occlusion of the fistula and one in the common bile duct for proper drainage of the biliary tract.

It is important to remember the need for appropriate preparation of the patient with invasive monitoring, jugular venous access, and general anesthesia, because any lapse during the procedure can be fatal. It is also essential to prepare a transparietal-hepatic access to the portal vein and the biliary tract with introducers that are compatible with the stents being inserted and with guidewires in position, as well as having the covered stents and balloon catheters already on the procedure table. Having readied all that, the endoscopist is authorized to remove the porto-duodenal stent and, immediately afterward, to perform portography in order to visualize the location and determine the grade of the fistula, as well as to localize the intrahepatic portal branches. After dilatation of the portal vein, the covered stent is immediately implanted in the vein, allowing the large fistula to be occluded. This is the same technique used when a stent is implanted in the portal vein for severe thrombosis or stenosis due to benign inflammatory processes or a malignant disease^(18)^.

**Figure 5:**
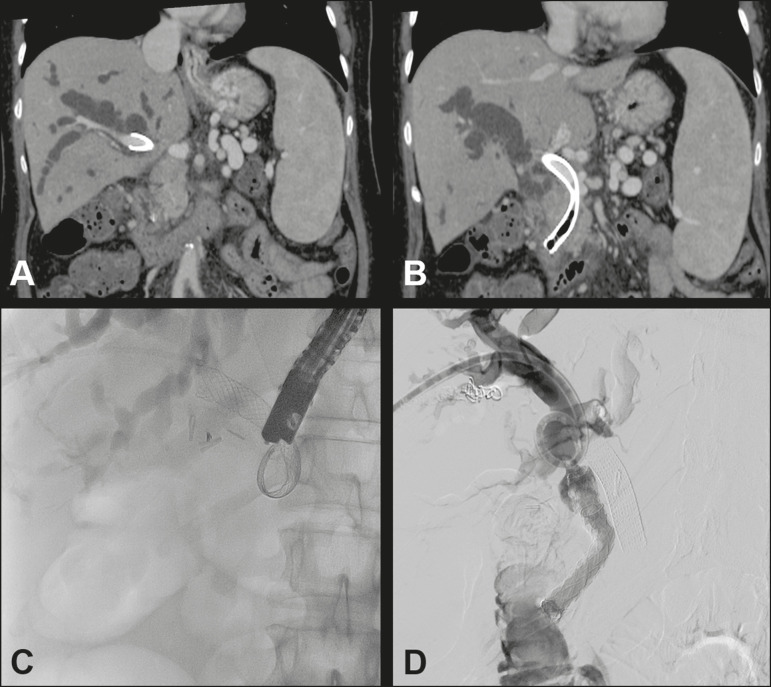
Female patient with locally advanced, inoperable pancreatic cancer. She underwent ERCP for the placement of a metal biliary stent, thereafter evolving to controlled hemorrhagic shock, followed by sepsis. Contrast-enhanced computed tomography of the abdomen and pelvis showed the proximal portion of the stent in the portal vein and its distal portion in the duodenum (**A,B**). We simultaneously performed procedures for percutaneous drainage of biliary ducts, removal of the porto-duodenal stent by ERCP, and percutaneous portal angioplasty for occlusion of the porto-duodenal fistula (**C,D**).

It is important to position the stent appropriately within in the portal vein, avoiding occlusion of intrahepatic portal branches (Figure 5). After the porto-duodenal fistula has been closed, the new biliary stent can be put in place.

## CONCLUSION

The percutaneous approach should be considered when the endoscopic intervention or reintervention fails. It is noteworthy that there are many options for managing failing plastic or metal biliary stents. Interventional radiologists should be familiar with the various relevant procedures and should know that such procedures can be performed successfully with a low rate of complications.
